# Factor B and C4b2a Autoantibodies in C3 Glomerulopathy

**DOI:** 10.3389/fimmu.2019.00668

**Published:** 2019-04-04

**Authors:** Jill J. Hauer, Dingwu Shao, Yuzhou Zhang, Carla M. Nester, Richard J. H. Smith

**Affiliations:** Molecular Otolaryngology and Renal Research Laboratories, University of Iowa, Iowa City, IA, United States

**Keywords:** complement dysregulation, autoantibodies, C3 glomerulopathies, factor B, C3 convertase, C5 convertase

## Abstract

C3 Glomerulopathy (C3G) is a renal disease mediated primarily by dysregulation of the alternative pathway of complement. Complement is the cornerstone of innate immunity. It targets infectious microbes for destruction, clears immune complexes, and apoptotic cells from the circulation, and augments the humoral response. In C3G, this process becomes dysregulated, which leads to the deposition of complement proteins—including complement component C3—in the glomerular basement membrane of the kidney. Events that trigger complement are typically environmental insults like infections. Once triggered, in patients who develop C3G, complement activity is sustained by a variety of factors, including rare or novel genetic variants in complement genes and autoantibodies that alter normal complement protein function and/or regulation. Herein, we review two such autoantibodies, one to Factor B and the other to C4b2a, the C3 convertase of the classical, and lectin pathways. These two types of autoantibodies are identified in a small fraction of C3G patients and contribute marginally to the C3G phenotype.

## Introduction

C3 Glomerulopathy (C3G) is an ultra-rare renal disease characterized by dysregulation of the alternative pathway (AP) of the complement cascade, which leads to the accumulation of complement cleavage products in the glomerular basement membrane (GBM) of the kidney. The complement cascade is comprised of over 30 proteins and can harness both the innate and adaptive immune systems to initiate and amplify an immune response in the presence of an antigen or unwanted cell debris. Activation occurs via three separate pathways: the classical, lectin, and alternative pathways. Classical pathway (CP) activation is mediated by the recognition of an antigen on a cell surface, the lectin pathway (LP) is initiated through the recognition of carbohydrates and other antigenic patterns, and the AP is spontaneously activated via the “tick-over” mechanism whereby an internal thioester bond of the C3 protein is hydrolyzed to form a functionally active form of the C3 protein (C3_H20_) (1, 2).

Once initiated, all pathways converge on the formation of the C3 convertase—an enzyme complex responsible for the amplification of the complement response—which rapidly cleaves and activates additional C3 protein. If complement activation is triggered through the AP, the AP C3 convertase is formed by the interaction of cleaved C3 and Factor B proteins (C3bBb). In contrast, if CP or LP activation occurs, the classical C3 convertase is formed (C4b2a). Cleavage of C3 by C4b2a leads to formation of the AP C3 convertase, C3bBb, and over 90% of continued complement activity continues through this convertase (3). Robust C3 convertase activity generates copious C3b and facilitates the formation of the C5 convertase, which marks the beginning of the terminal complement cascade. C5 activation by the C5 convertase leads to the formation of the membrane attack complex (MAC), which is ultimately responsible for inducing lysis of the identified pathogen (4, 5).

Rigorous regulation of complement is required to prevent unwanted complement activity. Cases of complement *dysregulation* lead to a diverse set of diseases underpinned by the common characteristic of damage to host tissue. C3 Glomerulopathy (C3G) is one such example (6–8). C3G is characterized by dysregulation of the AP, which leads to C3 deposition in the glomerulus. The diagnosis requires a renal biopsy, which by consensus must show by immunofluorescence the deposition of C3 in renal glomeruli that is at least 2-fold higher than any other immune reactant. Further categorization of C3G into subtypes is made by electron microscopy. If dense, intra-membranous, sausage-like deposits are seen, the diagnosis is dense deposit disease (DDD); when the deposits are lighter, cloud-like, and sub-epithelial, or sub-endothelial, the diagnosis is C3 Glomerulonephritis (C3GN) (9, 10). There is currently no disease-specific treatment for C3G and about 50% of patients progress to end stage renal disease (ESRD) within 10 years of diagnosis (11).

**Figure 1:**
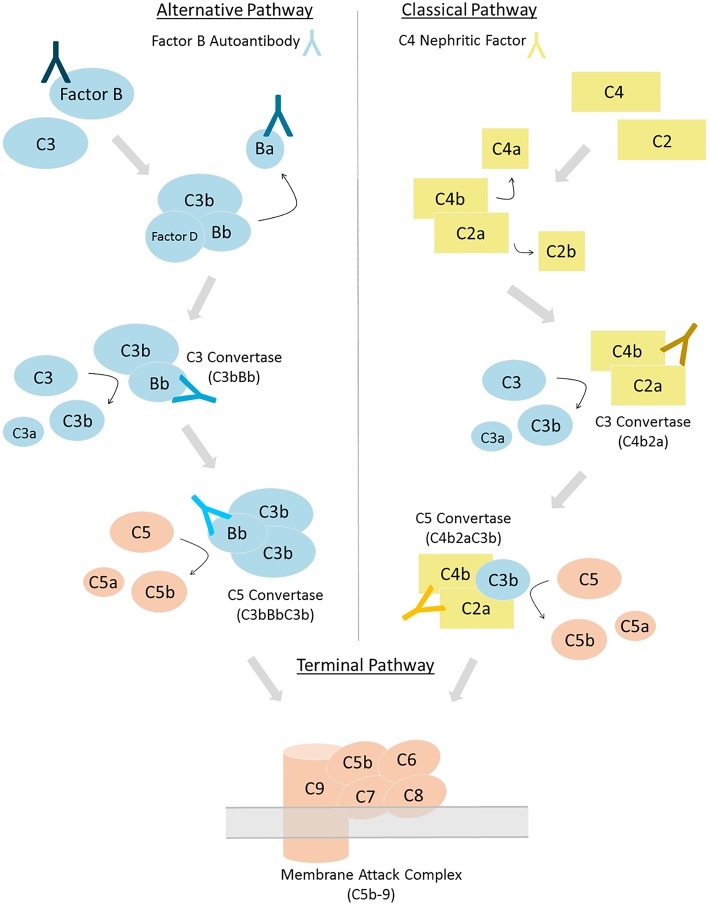
Complement can be activated via three distinct pathways. Factor B autoantibodies bind to Factor B and/or its protein fragments generated via alternative pathway activation (left). C4 nephritic factors bind to the classical and lectin pathway's C3 and C5 convertases (right). Differing shades of antibodies indicate possible epitopes; however, the epitope or epitopes of factor B autoantibodies and C4 nephritic factors may vary among patients.

The drivers of complement dysregulation in C3G can be identified in the majority of cases and include genetic variants in complement proteins and regulators, and/or autoantibodies specific to proteins or complexes of the complement cascade. The earliest description of autoantibodies that target complement proteins dates back to 1967 when C3- and C4-targeting immunoglobulins were described in the serum of several species of mammals upon stimulation with the animal's autologous, fixed complement components (12). Subsequently, autoantibodies have been described in virtually every branch of the complement system: autoantibodies targeting CP proteins (13, 14), LP proteins (15), AP proteins (16), protein complexes including the C3 (17), and C5 (18) convertases, and complement regulatory (19, 20) proteins. Autoantibodies to complement can be detected in a diverse spectrum of diseases including systemic lupus erythematosus (SLE), rheumatoid arthritis (RA), atypical hemolytic uremic syndrome (aHUS), and C3G (21). This review is tightly focused on two types of autoantibodies that are specific for Factor B and C4b2a (also known as C4 nephritic factors or C4Nefs). We discuss the current knowledge relevant to these two antibodies, methods for their robust detection, and their more recently appreciated role in C3G (Figure 1).

## Factor B Autoantibodies

Factor B autoantibodies (FBAAs) have been identified in a small number of individuals and exclusively in patients with C3G. The first report of FBAAs was in 2010—a DDD patient was positive for an IgG that bound the cleaved Bb fragment of Factor B and recognized an epitope on the C3 convertase (C3bBb). This antibody stabilized the C3 convertase and prevented its decay; it also prevented formation of the C5 convertase thereby inhibiting terminal pathway activity. Unlike the more common C3 nephritic factor, this FBAA did not bind a neoepitope on the C3 convertase and because it prevented terminal pathway activity, it tested negative in the hemolytic assay, which is traditionally used to detect the stabilizing effect of C3 nephritic factors (C3Nefs) on C3 convertase (16). In 2011, two more patients were reported to have FBAAs, which stabilized C3 convertase activity (22). All three patients were negative for C3Nefs (16, 22).

In a larger study on a cohort of 141 patients with C3G or Ig-associated membranoproliferative glomerulonephritis (immune complex glomerulonephritis, ICGN), seven patients were positive for FBAAs, three were positive for anti-C3b IgG, and five were positive for both FBAA and anti-C3b. Ten of these 15 patients were diagnosed with ICGN. Consistent with previous reports, the patients with FBAAs alone demonstrated specific enhancement of C3bBb activity only; there was no enhancement of C5 convertase activity. Patients who were positive for both FBAAs and anti-C3b antibodies showed enhancement of both C3 and C5 convertase activity. FBAA binding was mapped to the Bb fragment of Factor B in this study (23).

Taken together, these data suggest that FBAAs are present in only a small percentage of the C3G population and drive specific over-activity of C3 convertase. Factor B autoantibodies have not yet been associated with any other complement-mediated diseases.

## C4 Nephritic Factors

In 1979, two patients with partial lipodystrophy were reported to have a nephritic factor the activity of which was dependent on the presence of C2, thus implicating the involvement of the classical C3 convertase, C4b2a (24). Shortly thereafter, C4Nef was fully characterized in a patient with post-infectious glomerulonephritis (PIGN) and proposed to be a separate entity from C3Nef. In this study, C4Nef was demonstrated to be an IgG capable of stabilizing both cell-bound and fluid-phase classical C3 convertase (25). Further investigation has shown that C4Nefs protect the classical C3 convertase from decay mediated by CR1 and C4 binding protein (C4BP) but not from decay mediated by decay accelerating factor (DAF) (26–29).

In 1989, a study of 2 patients diagnosed with MPGN type I detected the presence of both C3Nefs and C4Nefs. Both patients had low circulating levels of C3 and C5 proteins, consistent with complement consumption due to the presences of these Nefs (30). A case study in 1993 of 100 hypocomplementemic patients with MPGN found that almost 20% were positive for C4Nefs. Half of these patients were also positive for C3Nefs. Interestingly, terminal complement pathway activity was only elevated in patients positive for both C3Nefs and C4Nefs (31). Another case report described an 18-year-old male with C3 deficiency who developed severe meningococcal meningitis. C3 consumption was driven by C4Nefs, which stabilized both C4b2a and C4b2aC3b, the C5 convertase of the classical pathway (32).

In aggregate, C4Nefs represent a possible cause of complement dysregulation in a small proportion of patients with complement-mediated diseases. Further studies are needed to elucidate their ability to stabilize both the C3 and C5 convertases of the classical pathway.

## Detection of FBAA and C4Nefs

**Figure 2:**
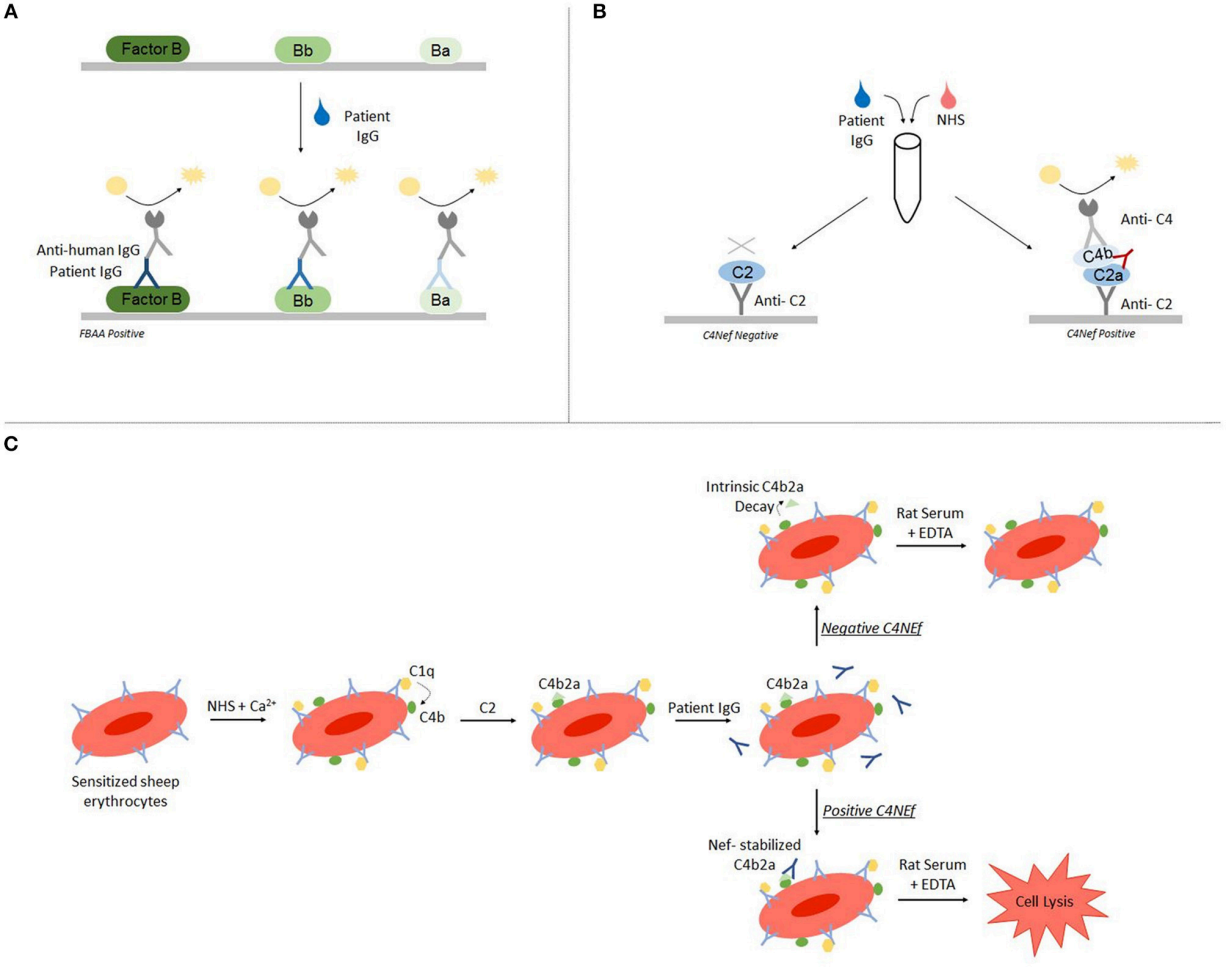
Autoantibody detection is performed using ELISA-based and hemolytic-based assays. Factor B autoantibodies can be detected using an ELISA-based method **(A)**. C4 nephritic factors can be detected using either a sandwich ELISA **(B)** or a hemolytic assay **(C)**.

Autoantibody detection in C3G is generally performed using ELISA and hemolytic-based assays. Both FBAA and C4Nefs have been identified using ELISA assays (23, 33, 34). For FBAAs, plates are coated with either the full length protein, the Bb fragment or the Ba fragment. After incubation with patient-purified IgG, binding can be detected using anti-human IgG (Figure 2A) (23). For C4Nef detection, a sandwich ELISA is used. This method indirectly detects C4Nefs by detecting the increased stabilization of the C4b2a complex. To form the CP C3 convertase (C4b2a), patient IgG is added to normal human serum (NHS). After ample time is allowed for intrinsic decay of C4b2a, the NHS-IgG mix is added to a plate coated with anti-C2 antibodies. Convertases that have not decayed (i.e., that have been stabilized by C4Nefs) are detected using an anti-C4 antibody (33). Both methods test binding of the autoantibody to its target complement protein, however neither measures functional activity (Figure 2B).

The hemolytic assay uses sheep erythrocytes, which are non-complement activators, to measure hemolytic activity (35). To detect C4Nefs, C4b2a is first built on sheep erythrocytes that have been antibody primed, and then the C4b2a-coated erythrocytes are incubated with patient IgG. After a fixed time during which convertase decay occurs in the absence of C4Nefs or is prevented in their presence, terminal complement components are added and lysis is quantitated. The amount of free heme reflects the degree of convertase stabilization and activity (33, 36). This assay provides an environment closer to *in vivo* conditions (Figure 2C).

Most recently, Blom et al. developed a modified hemolytic-based method that is sensitive not only to Nefs but also to genetic abnormalities (37). The method uses full patient serum with the addition of a C5 inhibitor to build the convertase on sheep or rabbit erythrocytes. This method is able to determine if Nefs are resistant to the extrinsic decay of complement regulatory proteins in full patient serum (36, 38).

Each of the above assays has limitations that should be recognized and are summarized in Table 1.

**Table 1:** Methods for detection of Factor B autoantibodies and C4 nephritic factors.

**Method**	**Autoantibody**	**Strengths**	**Limitations**
ELISA (Figure 2A)	Factor B	Ability to map domain of IgG binding epitope by coating ELISA plate with either FB, Bb, or Ba Test is inexpensive and fast	Anti-human IgG is used to detect FBAA binding—this tests for the presence of FBAA, but does not test the effect of FBAA on complement function Assay is performed using patient purified IgG and is therefore unable to test the effect of complement regulators on FBAA function Assay is performed on a protein-coated 96-well plate which does not represent *in vivo* conditions
ELISA (Figure 2B)	C4Nef	Assay is conducted with patient purified IgG and normal human serum—this means nef function is tested in the presence of complement regulatory proteins Test is inexpensive and fast	Anti-C2 and anti-C4 are used to capture the classical convertase—this means the presence of C4Nefs is indirectly tested, and does not discern function of the classical convertase Assay is performed on a protein-coated 96-well plate which does not represent *in vivo* conditions
Hemolytic (Figure 2C)	C4Nef	Assay is performed using sheep erythrocytes—a more realistic representation of *in vivo* conditions Convertase function is directly quantified by measuring hemolysis	Assay is technically difficult to perform Assay is performed using patient purified IgG and is therefore unable to test the effect of complement regulators on FBAA function Assay does not discern where on the convertase the C4Nef is binding

## The Consequence of FBAAs and C4Nefs in C3G

Many types of autoantibodies can be detected in patients with C3G. In aggregate, autoantibodies that stabilize and increase the half-life of the C3 and C5 convertases (C3Nefs and C5nefs, respectively) are identified in ~60% of patients (36, 39). Several studies have associated these autoantibodies with complement dysregulation, which supports the hypothesis that these autoantibodies are drivers of the C3G phenotype, although this relationship is not yet well understood.

Biomarker testing is often performed on sera from C3G patients to quantitate the degree of complement dysregulation. By measuring levels of circulating C3 and its cleavage products, for example, it is possible to identify when C3 convertase overactivity occurs. When low C3 levels (indicating C3 consumption, thus complement activation) are accompanied by low C5 and high soluble C5b-9 levels, dysregulation of both the C3 and C5 convertase is highly likely.

Data on the biomarker profile of C3G patients with FBAAs are anecdotal. Marinozzi et al. identified three patients diagnosed with C3G who were positive for FBAAs. The biomarker profile of these three patients, and nine additional patients with ICGN showed only an increase in the Bb fragment of cleaved Factor B (data summarized in Table 2) (23).

**Table 2:** Biomarker profiles associated with Factor B autoantibodies and C4 nephritic factors.

**Biomarker**	**FBAA (23)**	**C4Nef (40)**
C3	Normal	Low
C3c	Not Tested	High
FB	Not Tested	Normal
Ba	Not Tested	Normal
Bb	High	Normal
C2	Not Tested	Normal
C4	Not Tested	Normal
C4a	Not Tested	Normal
C5	Not Tested	Low
sC5b-9	Normal	High
Properdin	Not Tested	Low

In the most recent case series of 168 patients with C3G, five were positive for C4Nefs using the hemolytic-based assay. Of note, two of the five patients positive for C4Nefs were also positive for other autoantibodies—one patient was positive for C3Nefs and the other for Factor H autoantibodies. All five patients showed a biomarker profile consistent with dysregulation of the C3 and C5 convertase (findings summarized in Table 2) (40). These data are consistent with a study by Blom et al. who reported an individual with C3G positive for C4Nefs (41).

## Conclusions

C3G is a complex disease, making a composite view of complement biomarkers and complement function, coupled with a genetic analysis and a thorough screen for autoantibodies essential in every patient. FBAAs and C4Nefs drive disease in a small fraction of patients. The small numbers of patients positive for these autoantibodies makes it difficult to discern the magnitude of their role as a disease driver, but as testing for these autoantibodies becomes routine, their impact will become better defined. More precise assays, which measure not only epitopes and function but also provide data on concentration and convertase avidity, are needed to better understand the nuanced impact of these autoantibodies on disease course and outcome.

## Author Contributions

JH, DS, YZ, CN, and RS contributed to the conception and design of the study. DS and YZ provided protocols for detection methods. JH performed the literature review. JH and RS wrote the manuscript. All authors contributed to manuscript revision, read, and approved the submitted version.

### Conflict of Interest Statement

RS is the director and CN is the associate director of the MORL, which offers testing of complement function in patients with ultra-rare complement -mediated renal diseases. The remaining authors declare that the research was conducted in the absence of any commercial or financial relationships that could be construed as a potential conflict of interest.
